# Exercise Training Restores Cardiac Protein Quality Control in Heart Failure

**DOI:** 10.1371/journal.pone.0052764

**Published:** 2012-12-27

**Authors:** Juliane C. Campos, Bruno B. Queliconi, Paulo M. M. Dourado, Telma F. Cunha, Vanessa O. Zambelli, Luiz R. G. Bechara, Alicia J. Kowaltowski, Patricia C. Brum, Daria Mochly-Rosen, Julio C. B. Ferreira

**Affiliations:** 1 Department of Anatomy, Institute of Biomedical Sciences, University of Sao Paulo, Sao Paulo, Brazil; 2 Departamento de Bioquímica, Instituto de Química, Universidade de São Paulo, Sao Paulo, Brazil; 3 Heart Institute, University of Sao Paulo, Sao Paulo, Brazil; 4 School of Physical Education and Sport, University of Sao Paulo, Sao Paulo, Brazil; 5 Butantan Institute, Sao Paulo, Brazil; 6 Department of Chemical and Systems Biology, Stanford University School of Medicine, Stanford, California, United States of America; Instituto de Investigación Hospital 12 de Octubre, Spain

## Abstract

Exercise training is a well-known coadjuvant in heart failure treatment; however, the molecular mechanisms underlying its beneficial effects remain elusive. Despite the primary cause, heart failure is often preceded by two distinct phenomena: mitochondria dysfunction and cytosolic protein quality control disruption. The objective of the study was to determine the contribution of exercise training in regulating cardiac mitochondria metabolism and cytosolic protein quality control in a post-myocardial infarction-induced heart failure (MI-HF) animal model. Our data demonstrated that isolated cardiac mitochondria from MI-HF rats displayed decreased oxygen consumption, reduced maximum calcium uptake and elevated H_2_O_2_ release. These changes were accompanied by exacerbated cardiac oxidative stress and proteasomal insufficiency. Declined proteasomal activity contributes to cardiac protein quality control disruption in our MI-HF model. Using cultured neonatal cardiomyocytes, we showed that either antimycin A or H_2_O_2_ resulted in inactivation of proteasomal peptidase activity, accumulation of oxidized proteins and cell death, recapitulating our *in vivo* model. Of interest, eight weeks of exercise training improved cardiac function, peak oxygen uptake and exercise tolerance in MI-HF rats. Moreover, exercise training restored mitochondrial oxygen consumption, increased Ca^2+^-induced permeability transition and reduced H_2_O_2_ release in MI-HF rats. These changes were followed by reduced oxidative stress and better cardiac protein quality control. Taken together, our findings uncover the potential contribution of mitochondrial dysfunction and cytosolic protein quality control disruption to heart failure and highlight the positive effects of exercise training in re-establishing cardiac mitochondrial physiology and protein quality control, reinforcing the importance of this intervention as a non-pharmacological tool for heart failure therapy.

## Introduction

Heart failure is a common endpoint of most cardiovascular diseases and a leading cause of morbidity and mortality worldwide. There is a consensus that adjuvant therapies for cardiovascular disease are able to increase patients' quality of life and survival rates [1]. Acting as a non-pharmacological therapy, exercise training reduces a number of cardiovascular risk factors [2], [3] and has been recognized as an important and safe strategy for preventing and treating heart failure [4], [5], [6]. However, the underlying cellular mechanisms by which exercise training improves heart failure patients' clinical outcome are still under investigation.

Mitochondrial dysfunction has been widely recognized as key player in the progression of cardiovascular diseases [7]. Exacerbated generation of reactive oxygen species (ROS) due to impaired mitochondrial bioenergetics is believed to underlie intra- and extra-mitochondrial signal transduction during cardiac remodeling and heart failure [8]. Evidences indicate that 4-hydroxy-2-nonenal (4-HNE), a long-lived lipid peroxidation product that accumulates during oxidative stress, irreversibly interacts with and inactivates mitochondrial, cytosolic and membrane proteins [9], [10], [11]. However, the contribution of 4-HNE protein adduction to heart failure pathophysiology remains elusive.

Over the last years, *in vitro* studies have demonstrated that specific subunits of the 20S proteasome are targeted for modification by 4-HNE, which results in reduced proteasomal peptidase activities [9], [10]. Recent findings showing that the ubiquitin–proteasome system is negatively regulated by oxidative stress during cardiac ischemia-reperfusion injury generated the hypothesis that mitochondrial dysfunction-mediated redox imbalance may negatively affect ubiquitin-proteasome activity in an ATP-independent manner.

Over the past decades, the ubiquitin–proteasome system has emerged as an important player on essential cellular processes such as cellular proliferation, differentiation and apoptosis [12], [13], [14]. The ubiquitin–proteasome system is the primary effector of the protein quality control process, protecting long-lived cells, such as neurons and cardiomyocytes, through selective removal of polypeptides that are terminally misfolded and toxic to the cell [15]. Perturbations in the ubiquitin–proteasome system have been shown to disturb protein turnover and thereby affect cell function. Recent studies have highlighted the role of the ubiquitin-proteasome system in stressed cardiac phenotypes including cardiac remodelling and heart failure [16]. Accumulation of ubiquitinated and damaged proteins is a common feature of human failing hearts and indicates the relevance of the ubiquitin–proteasome system in maintaining cardiac homeostasis [16], [17]. However, it remains to be determined whether dysfunction of specific protein quality control components, such as the ubiquitin–proteasome system, contributes to the development of heart failure, and which signaling events regulate them.

Despite the increased knowledge regarding the molecular basis of mitochondrial ROS generation and redox balance in cardiovascular diseases, there is still uncertainty in the literature regarding the contribution of the long-lived lipid peroxidation product 4-HNE in protein modification and intracellular system disruption in cardiovascular diseases. Some evidence indicates that 4-HNE modification of proteins is triggered by mitochondrial dysfunction and provides an operational criterion for inactivation of mitochondrial and cytosolic systems related to cell survival [18], [19]. Therefore, the objectives of the study were: 1) to test the hypothesis that mitochondrial dysfunction disrupts cardiac protein quality control through specific 4-HNE modification/inhibition of proteasome and 2) to verify whether exercise training prevents impairment of mitochondrial metabolism, 4-HNE adduct formation and protein quality control disruption in heart failure.

## Materials and Methods

### Study design

**Figure 1:**
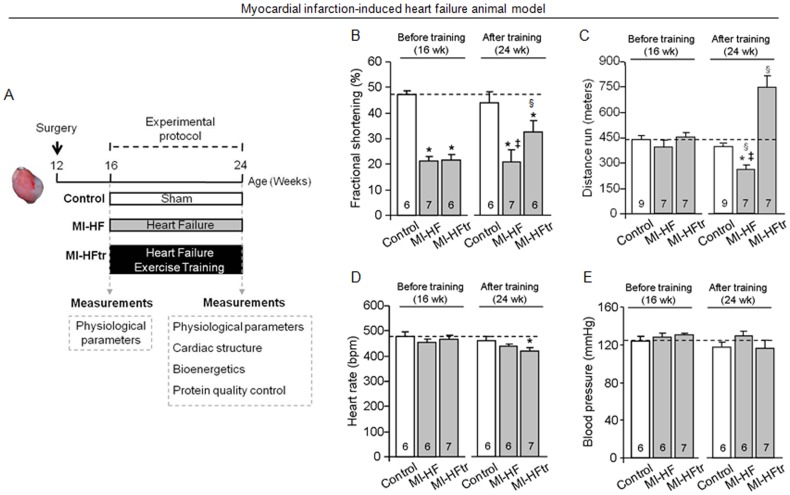
Exercise training improves cardiac function and exercise tolerance in myocardial infarction-induced heart failure. Schematic panel (A). Fractional shortening (B), distance run (C), heart rate (D) and blood pressure (E) in control (sham, white bars), MI-HF (gray bars) and MI-HF exercise trained (MI-HFtr, gray bars) rats before and after 8 wks of either sedentary or exercise training protocol. Error bars indicate SEM. Interaction between main effects: fractional shortening [F (2, 15) = 5.28, p = 0.0183]; distance run [F (2, 31) = 48.97, p<0.0001]; heart rate [F (2, 13) = 4.06, p = 0.0425] and blood pressure [F (2, 13) = 1.05, p = 0.3764]. §, p<0.05 vs. before experimental protocol. *, p<0.05 vs. control (sham) rats. ‡, p<0.05 vs. MI-HFtr rats.

The present investigation was carried out in male Wistar rats assigned into three experimental groups: sham (control, n = 9), myocardial infarction-induced heart failure (MI-HF, n = 10) and exercised-trained MI-HF (MI-HFtr, n = 8). Heart failure was induced by myocardial infarction surgery. Four weeks later, physiological parameters were determined and animals with heart failure were randomly assigned into sedentary (MI-HF) and exercise-trained (MI-HFtr) groups (Figure 1A). MI-HFtr rats performed a moderate-intensity running training on a motor treadmill over eight weeks (from 4^th^–6^th^ month of age). At the end of the protocol, physiological parameters were re-analysed. Forty-eight hours later, all rats were killed by decapitation and cardiac structure, bioenergetics and protein quality control measurements were performed.

### Animals and procedures

A cohort of male Wistar rats (250–300 g) was selected for the study. Rats were maintained in a 12∶12 h light-dark cycle and temperature-controlled environment (22°C) with free access to standard laboratory chow (Nuvital Nutrientes, Curitiba, PR Brazil) and tap water. This study was conducted in accordance with the ethical principles in animal research adopted by the Brazilian College of Animal Experimentation (www.cobea.org.br). The animal care and protocols in this study were reviewed and approved by the Ethical Committee of Medical School of University of São Paulo (2008/40).

### Myocardial infarction-induced heart failure model

Myocardial infarction was induced by ligation of the left anterior descending coronary artery (LAD), as previously described [20]. We have chosen this model since myocardial infarction is the underlying etiology of heart failure in nearly 70% of patients [21]. Male Wistar rats were anesthetized with ketamine (50 mg^.^kg^−1^ IP) and xylazine (10 mg^.^kg^−1^ IP), endotracheally intubated, and mechanically ventilated with room air (respiratory rate of 60–70 breaths/min and tidal volume of 2.5 mL). Left thoracotomy between the fourth and fifth ribs was performed and the LAD was ligated. After the surgery, animals were monitored daily. Heart failure was observed four weeks after coronary artery ligation and was defined when animal presented pathological cardiac remodeling accompanied by left ventricle dysfunction, cardiac dilation and exercise intolerance (Tables 1 and 2), according to the Guidelines of American Heart Association [22]. Left thoracotomy with equal procedure duration to that of heart failure group, but without LAD ligation, was undertaken in the sham group (control).

**Table 1:** Physiological parameters.

	Before experimental protocol (16 wk)			After experimental protocol (24 wk)€		
Parameter	Control (9)	MI-HF (7)	MI-HFtr (7)	Control (9)	MI-HF (7)	MI-HFtr (7)
Peak VO_2_, mL O_2_·kg^−1^·min^−1^	65.6±1.8	67.5±2.4	68.4±3.2	58.7±2.5	47.8±2.4	66.3±3.1
BW, g	418±12	390±11	391±11	432±22	429±14	415±12
CS activity, µmol·mg^−1^·min^−1^	-	-	-	4476±718	3200±677*‡	6386±1007*
HW/BW, mg·g^−1^	-	-	-	2.0±0.1	2.4±0.1*	2.4±0.1*
MI area, %	-	-	-	-	31±3	35±4
Cardiomyocyte width, µm	-	-	-	14.33±0.17	14.90±0.17*	14.42±0.22
Cardiac collagen content, %	-	-	-	5.45±0.33	6.77±0.44*‡	5.66±0.32

Peak VO_2_ (in mL O_2_·kg^−1^·min^−1^), body weight (BW in grams), *soleus* muscle citrate synthase activity (CS in µmol·mg^−1^·min^−1^), heart weight/body weight ratio (HW/BW), myocardial infarction (MI) area, cardiomyocyte width (µm) and cardiac collagen content (%) data in control (sham), MI-HF and MI-HF exercise trained (MI-HFtr) rats (Mean ± SEM).

€ Main time effect: peak VO_2_ [F (1, 18) = 9.75, p = 0.0058] pre-training values>post-training values and BW [F (1, 16) = 10.73, p = 0.0047]. CS activity [F (2, 21) = 29.80, p<0.0001] *MI-HF<control (p = 0.0047) and ‡MI-HFtr (p<0.0001), *MI-HFtr>control (p = 0.0002); HW/BW [F (2, 16) = 8.55, p = 0.0029] *control<MI-HF (p = 0.0044) and MI-HFtr (p = 0.0036); cardiomyocyte width [F (2, 14) = 11.42, p<0.0001] *MI-HF>control (p<0.0001) and cardiac collagen content [F (2, 23) = 3.76, p = 0.0245] *MI-HF>control (p = 0.0189) and ‡MI-HFtr (p = 0.0311).

**Table 2:** Echocardiographic measurements.

	Before experimental protocol (16 wk)			After experimental protocol (24 wk)€		
Parameter	Control (9)	MI-HF (7)	MI-HFtr (7)	Control (9)	MI-HF (7)	MI-HFtr (7)
EF, %	83.2±1.22	47.2±3.89*	48.0±4.43*	79.2±3.45	47.0±5.04*‡	64.3±6.16*^§^
IVSd, mm	1.4±0.02	1.2±0.08	1.2±0.06	1.5±0.05	1.2±0.05	1.4±0.11
IVSs, mm	2.5±0.10	1.3±0.10	1.3±0.09	2.6±0.13	1.5±0.13	2.0±0.29
LVEdD, mm	7.0±0.14	9.1±0.27	8.7±0.21	7.9±0.29	9.1±0.13	8.7±0.4
LVEsD, mm	3.7±0.14	7.2±0.24	6.8±0.23	4.5±0.37	7.2±0.51	6.0±0.63
LVPWd, mm	1.4±0.03	1.6±0.09	1.5±0.13	1.6±0.08	1.3±0.06^§^	1.5±0.12
LVPWs, mm	2.6±0.14	2.6±0.17	2.7±0.20	2.7±0.18	2.2±0.13	2.7±0.24

Left ventricular ejection fraction (EF), interventricular septum in diastole (IVSd), interventricular septum in systole (IVSs), left ventricular end-diastolic diameter (LVEdD), left ventricular end-systolic diameter (LVEsD), left ventricular posterior wall in diastole (LVPWd) and left ventricular posterior wall in systole (LVPWs) were obtained before and after 8 wks of the experimental protocol in control (sham), MI-HF and MI-HF exercise trained (MI-HFtr) rats (Mean ± SEM). Interaction between main effects: EF [F (2, 15) = 6.84, p = 0.0077] *control>MI-HF (p<0.0001) and MI-HFtr (p<0.0001) before and after experimental protocol, ‡MI-HFtr>MIHF after experimental protocol (p = 0.0136) and §MI-HFtr before<after experimental protocol (p = 0.0167); and LVPWd [F (2, 15) = 4.52, p = 0.0289] §MI-HF before>after experimental protocol (p = 0.0401).

€ Main time effect: IVSs [F (1, 15) = 5.98, p = 0.0272] pre-training values<post-training values.

### Graded treadmill exercise test and oxygen uptake measurement

After being adapted to treadmill exercises and the test environment for over one week (10 minutes each session), rats were placed on the exercise streak inside a metabolic chamber equipped with plastic tubes that allowed air into the chamber and out to a high-resolution oxygen analyzer (FC-10, Sable Systems International, Las Vegas, USA). Ambient air was pumped into the chamber (3500 mL^.^min^−1^), continuously extracted at the same rate and directed to the oxygen analyzer. Oxygen fraction in effluent air was registered every second. The analyzer was calibrated with known gas mixtures every day of tests. Each rat had a twenty-minute rest period and a ten-minute warm-up at 3 m^.^min^−1^ before the test protocol. Treadmill speed was increased by 3 m^.^min^−1^ every 3 minutes until the animal was unable to run [23]. We considered the VO_2_ reached at the highest workload during the treadmill test as peak VO_2_. It is worth mentioning that peak VO_2_ has been described to the best predictor of mortality in humans with cardiovascular diseases [24]. VO_2_ (mL O_2_^.^kg^−1.^min^−1^) was calculated using the measured flow through the metabolic chamber (3500 mL^.^min^−1^), expired fraction of effluent oxygen (E), fraction of oxygen in room air (A) and rat body mass (M [kg]), as described by the formula: VO_2_ = [3500×(A−E)]/M. Peak VO_2_ was measured both before (week 16) and after experimental protocol (week 24) (Figure 1).

### Running training protocol

Heart failure rats performed moderated-intensity running training on a motor treadmill, 5 days/wk, 60 min/day. Running speed and duration of exercise were progressively increased to elicit 60% of maximal speed at the second week of training, corresponding to the maximal lactate steady state workload [25]. At the fourth week of training, run capacity was evaluated in order to readjust exercise training intensity. Treadmill running skills were maintained in sedentary HF and sham rats by treadmill running for 5 min, twice a week. This procedure was performed in order to avoid any interference of treadmill stress on the variables studied. This latter activity did not seem to alter maximal exercise capacity (Table 1).

### Cardiovascular measurements

Heart rate and blood pressure were determined noninvasively using a computerized tail-cuff system (BP 2000 Visitech Systems) described elsewhere [26]. Rats were acclimatized to the apparatus during daily sessions over 4 days, one week before starting the experimental period.

Non-invasive cardiac function evaluation was performed by M-mode echocardiography in anesthetized (isoflurane 3%) sham and HF rats, four and twelve weeks after surgery. Briefly, rats were positioned in the supine position with front paws wide open and ultrasound transmission gel was applied to the precordium. Transthoracic echocardiography was performed using an Acuson Sequoia model 512 echocardiographer equipped with a 14-MHz linear transducer. Left ventricle systolic function was estimated by fractional shortening (FS) as follows: FS(%) = [(LVEDD−LVESD)/LVEDD]×100, where LVEDD is the left ventricular end-diastolic diameter, and LVESD is the left ventricular end-systolic diameter.

### Cardiac structural analysis

Forty-eight hours after the end of protocol, all rats were killed by decapitation and their tissues were harvested. Cardiac chambers were then fixed by immersion in 4% buffered formalin and embedded in paraffin for routine histological processing. Sections (4 µm) were stained with Hematoxylin-eosin, Picrosirius red or Masson's trichrome for the quantification of the cardiomyocyte width, cardiac collagen content and myocardial infarct area, respectively. These measurements were performed in the left ventricle free wall with a computer-assisted morphometric system (Leica Quantimet 500, Cambridge, UK, England), as described previously [27]. The myocardial infarcted area was expressed as a percentage of total surface area of the left ventricle [28].

### Citrate synthase activity

Soleus muscles were homogenized in phosphate buffer (50 mM sodium phosphate, 1 mM EDTA and protease inhibitor cocktail (Sigma-Aldrich), pH 7.4) and centrifuged for 15 minutes at 12000 g and 4°C, pellet was discarded and supernatant was used for the assay. Assay mixture contained 100 mM Tris, 1 mM EDTA, 0.2 mM DTNB, 0.1 mM acetil-CoA, 1% (v∶v) Triton X-100, sample (130 µg of soluble proteins per mL of total assay) and 0.5 mM oxaloacetate (added latest), as originally described [29]. Sample absorbance was monitored at 412 nm in 96-well plate during 10 minutes at 25°C and maximal citrate synthase activity was measured within the linear range of the assay.

### Mitochondrial isolation

Heart mitochondria were isolated as described elsewhere [30]. Briefly, cardiac samples from a remote area were minced and homogenized in isolation buffer (300 mM sucrose, 10 mM Hepes, 2 mM EGTA, pH 7.2, 4°C) containing 0.1 mg^.^mL^−1^ of type I protease (bovine pancreas) to release mitochondria from within muscle fibers and later washed in the same buffer in the presence of 1 mg^.^mL^−1^ bovine serum albumin. The suspension was homogenized in a 40 mL tissue grinder and centrifuged at 950 g for 5 min. The resulting supernatant was centrifuged at 9500 g for 10 min. The mitochondrial pellet was washed, resuspended in isolation buffer and submitted to a new centrifugation (9500 g for 10 min). The mitochondrial pellet was washed and the final pellet was resuspended in a minimal volume of isolation buffer.

### Mitochondrial H_2_O_2_ release

Mitochondrial H_2_O_2_ release was measured as described elsewhere [31]. Briefly, mitochondrial H_2_O_2_ release was measured in a 0.125 mg protein^.^mL^−1^ mitochondrial suspensions in buffer containing 125 mM sucrose, 65 mM KCl, 10 mM Hepes, 2 mM inorganic phosphate, 2 mM MgCl_2_, 100 µM EGTA and 0.01% bovine serum albumin, pH 7.2, at 30°C, with continuous stirring. Amplex Red (25 µM) oxidation was followed in the presence of 0.5 U^.^mL^−1^ horseradish peroxidase and using succinate, malate and glutamate (2 mM of each) as substrates. Amplex Red is oxidized in the presence of extramitochondrial horseradish peroxidase bound to H_2_O_2_, generating resorufin, which can be detected using a fluorescence spectrophotometer. Excitation/emission wavelengths were 563/587 nm. Calibration was conducted by adding H_2_O_2_ at known concentrations (A_240_ = 43.6 M^−1.^cm^−1^) to the experimental buffer.

### Mitochondrial O_2_ consumption

Mitochondrial O_2_ consumption was monitored in a 0.25 mg protein^.^mL^−1^ mitochondrial suspension under the same conditions as H_2_O_2_ release measurements using a computer-interfaced Clark-type electrode (OROBOROS Oxygraph-2k) operating with continuous stirring at 37°C [31]. Succinate, malate and glutamate (2 mM of each) were used as substrates and ADP (1 mM) was added to induce State 3 respiratory rate. A subsequent addition of oligomycin (1 µg^.^mL^−1^) was used to determine State 4 rate. Respiratory control ratio (RCR) was calculated by dividing State 3 by State 4 oxygen consumption rates, which demonstrates the tightness of the coupling between mitochondrial respiration and phosphorylation.

### Maximal mitochondrial calcium uptake

Extramitochondrial Ca^2+^ concentrations were measured in a 0.125 mg protein^.^mL^−1^ mitochondrial suspensions using the fluorescent probe Calcium Green (100 nM) as described [32]. The reactions were carried out under the same conditions as H_2_O_2_ release measurements with continuous stirring at 37°C. For each experiment, consecutive additions of 50 µM CaCl_2_ were made until the mitochondria failed to reduce extramitochondrial Ca^2+^. We therefore plotted a calibration curve that correlates fluorescence and Ca^2+^ concentration. Succinate, malate and glutamate (2 mM of each) were used as substrates and 100 µM EGTA was used to establish the baseline. Excitation/emission wavelengths were 506/532 nm.

### In vitro 4-hydroxy-2-nonenal modification of proteasome

Either 50 µg of heart lysate from control (*sham*) animals or 2 µg of purified 20S proteasome (PW8720, Enzo Lif Sci, PA) were incubated with different concentrations of 4-HNE (10 or 100 µM) in assay buffer containing 25 mM Tris-HCl, 1 mM CaCl_2_, 20 mM MgCl_2_, pH 7.5 at 37°C for 60 minutes. Dithiothreitol (DTT, 1 µM) was added to the reaction either 30 minutes prior or after 4-HNE incubation to assess the reversibility of 4-HNE modification of proteasome. Measurement of proteasome activity was carried out after finishing the *in vitro* assay.

### Proteasome activity

ATP-dependent chymotrypsin-like activity of the proteasome was assayed in the total lysate from heart, isolated cardiomyocyte or purified proteasome using the fluorogenic peptide Suc-Leu-Leu-Val-Tyr-7-amido-4-methylcoumarin (LLVY-AMC, 25 µM). The assay was performed in a microtiter plate (FlexStation II 384, Molecular Device Inc, CA), in assay buffer containing 25 mM Tris-HCl, 5.0 mM MgCl_2_, 25 µM ATP, pH 7.5. Kinetic analyses were carried out using 50 µg of protein for 30 min at 37°C in the presence and absence of 1 µM epoxomicin (a selective proteasome inhibitor), with the difference attributed to ATP-dependent proteasomal activity. Excitation/emission wavelengths were 350/440 nm. Proteasome activity was linear for 30 min under the conditions of the assays.

### Cell culture

Cardiac myocytes were isolated from 1-day-old Sprague-Dawley rat litters, as described [33].

### Cell death

Cell death was measured using the cytotoxicity detection kit (Roche), which measures LDH released in the medium. Percentage cytotoxicity was calculated according to the manufacturer's instructions.

### Immunoprecipitation

Total lysate of rat heart (500 µg protein) was incubated with the indicated antibodies for 3 h at 4°C, followed by incubation with protein A/G agarose beads (Santa Cruz Biotechnology) for 1 h at 4°C. The immunoprecipitates were separated on SDS–PAGE and transferred onto nitrocellulose membranes. The membranes were then probed with the indicated antibodies.

### Western blot

20S proteasome (α5/α7, β1, β5, β7 subunits), polyubiquitinated proteins, soluble oligomers, HSP25, αβ-crystallin and 4-HNE expression levels were evaluated by western blotting in total extracts from the ventricular remote area. Briefly, samples were subjected to SDS-PAGE in polyacrylamide gels (6–15%) depending upon protein molecular weight. Cardiac soluble oligomers levels were evaluated in non-denaturating gel electrophoresis according to Glabe et al. (2004) [34]. After electrophoresis, proteins were electrotransferred to nitrocellulose membranes (BioRad Biosciences; Piscataway, NJ, USA). Equal gel loading and transfer efficiency were monitored using 0.5% Ponceau S staining of blot membrane. Blotted membrane was then blocked (5% nonfat dry milk, 10 mM Tris-HCl (pH = 7.6), 150 mM NaCl, and 0.1% Tween 20) for 2 h at room temperature and then incubated overnight at 4°C with specific antibodies against 20S proteasome (α5/α7, β1, β5, β7 subunits) and polyubiquitinated proteins (Biomol Int., PA, USA), HSP25 and αβ-crystallin (Stressgen, MI, USA), 4-HNE (Calbiochem, HE, Germany), GAPDH (Advanced Immunochemical Inc. CA, USA) and soluble oligomers A11 (Invitrogen, CA, USA). Binding of the primary antibody was detected with the use of peroxidase-conjugated secondary antibodies (rabbit or mouse, depending on the protein, for 2 h at room temperature) and developed using enhanced chemiluminescence (Amersham Biosciences, NJ, USA) detected by autoradiography. Quantification analysis of blots was performed with the use of Scion Image software (Scion based on NIH image). Samples were normalized to relative changes in GAPDH and expressed as percent of control.

### Cellular oxidized proteins

Protein oxidation was determined as previously described [35]. The carbonyl groups in the protein side chains were derivatized to 2,4-dinitrophenylhydrazone (DNPhydrazone) by reaction with 2,4-dinitrophenylhydrazine (DNPH). The DNP-derivatized protein samples were separated by polyacrylamide gel electrophoresis followed by Western blotting.

### Statistical analysis

**Figure 2:**
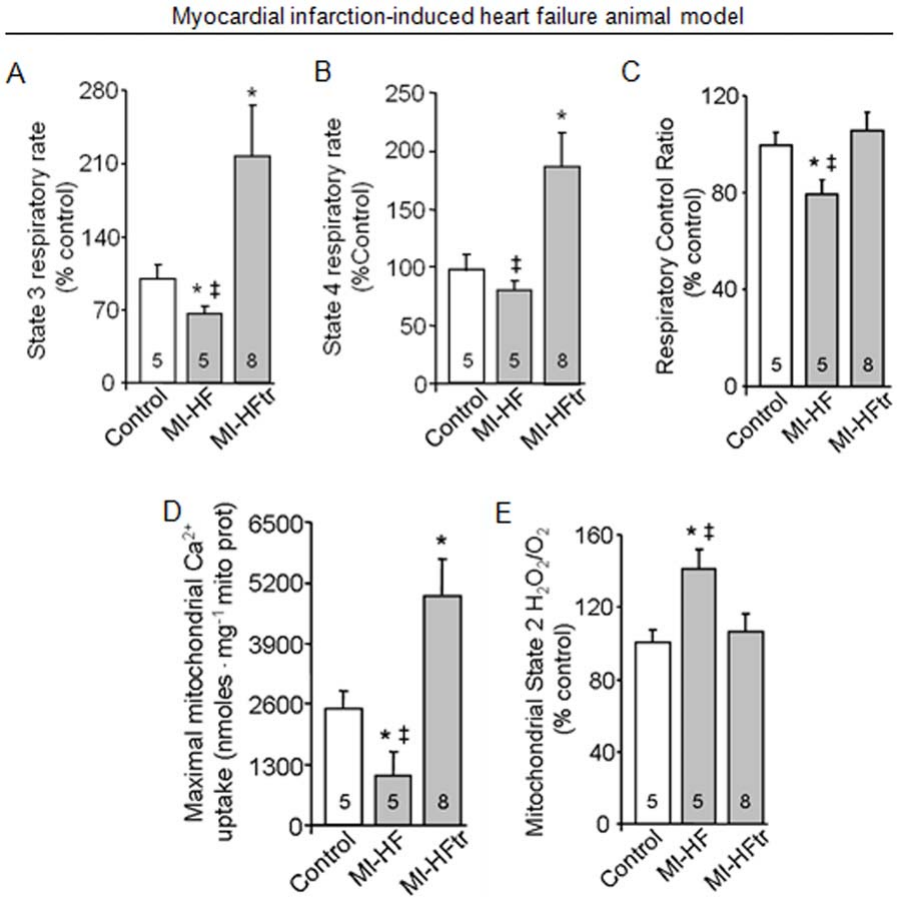
Exercise training improves oxygen consumption and reduces H_2_O_2_ release in cardiac isolated mitochondria from myocardial infarction-induced heart failure animal. Mitochondrial state 3 (A) and state 4 (B) respiratory rates; respiratory control ratio (C); maximum calcium uptake (D) and H_2_O_2_ release (E) in heart samples from 24 week-old control (sham, white bars), MI-HF (gray bars) and MI-HF exercise trained (MI-HFtr, gray bars) rats. All measurements were performed in the ventricular remote area. Error bars indicate SEM. Mitochondrial state 3 [F (2, 41) = 8.62, p = 0.0007] and state 4 [F (2, 41) = 8.86, p = 0.0006] respiratory rates; respiratory control ratio [F (2, 45) = 3.26, p = 0.0475]; maximum calcium uptake [F (2, 14) = 5.72, p = 0.0152] and H_2_O_2_ release [F (2, 37) = 5.28, p = 0.0095]. *, p<0.05 vs. control (sham) rats. ‡, p<0.05 vs. MI-HFtr rats.

**Figure 3:**
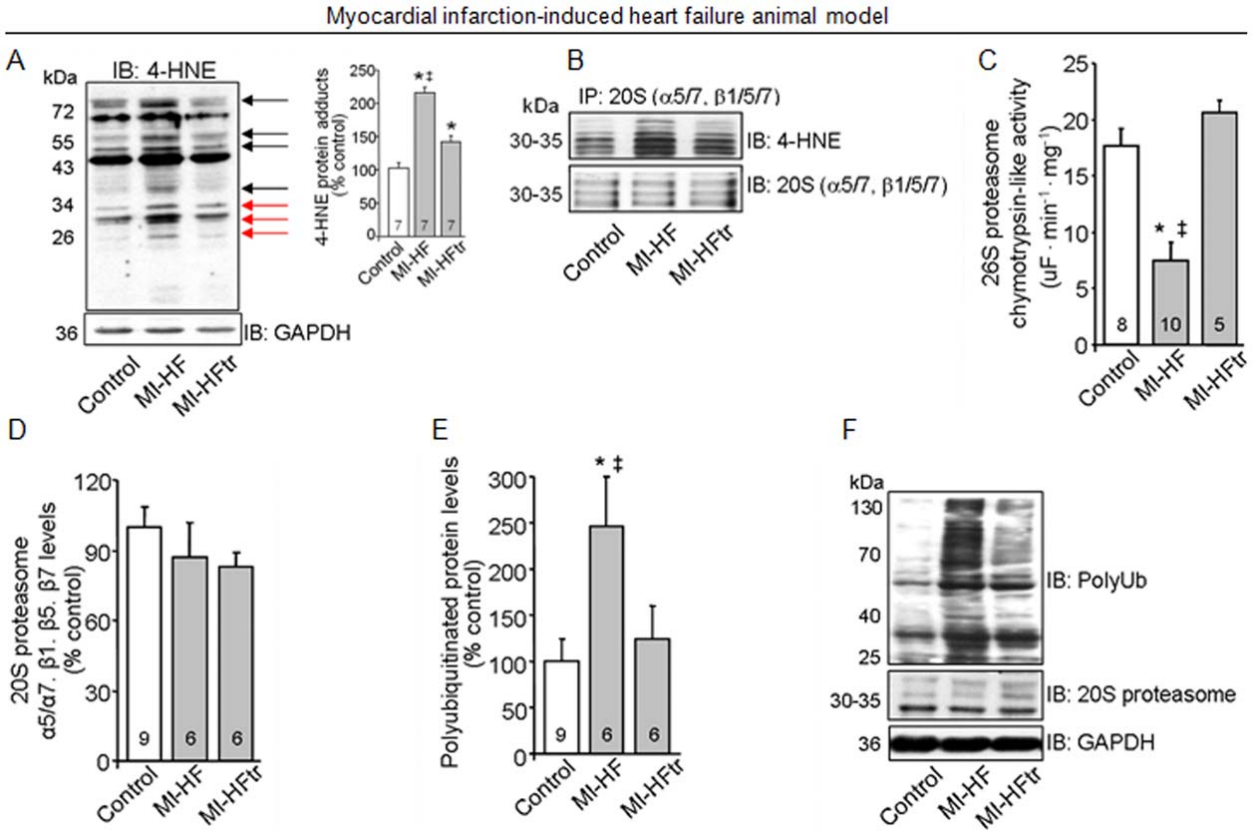
Exercise training decreases 4-HNE modification of proteasome and re-establishes cardiac ubiquitin-proteasome system function in myocardial infarction-induced heart failure. (A) 4-HNE protein adducts in heart samples from 24 week-old control (sham), MI-HF and MI-HF exercise trained (MI-HFtr) rats. Protein expression was normalized by GAPDH. Inset: Representative blot of 4-HNE protein adducts. Black arrows indicate changes in the adduct formation in MI-HF and MI-HFtr samples, red arrows indicate changes in the adduct formation of proteins at the molecular weight of proteasomal subunits. (B) 20S proteasome subunits (α5/α7, β1, β5, β7) were precipitated from left ventricle tissue from 24-week-old control, MI-HF and MI-HFtr rats (B, n = 3 per group), and then probed with 4-HNE-modified proteins antibody. Equal sample loading was verified using α5/α7, β1, β5 and β7 proteasome subunits antibody. (C) Chymotrypsin-like activity of 26S proteasome, (D) 20S proteasome α5/α7, β1, β5, β7 protein levels and (E) polyubiquitinated proteins levels in heart samples from 24 week-old control, MI-HF and MI-HFtr rats. Protein expression was normalized by GAPDH. (F) Representative blots of polyubiquitinated proteins, 20S proteasome and GAPDH. All measurements were performed in the ventricular remote area. Error bars indicate SEM. 4-HNE protein adducts [F (2, 15) = 42.58, p<0.0001]; chymotrypsin-like activity of 26S proteasome [F (2, 25) = 12.90, p = 0.0001]; 20S proteasome α5/α7, β1, β5, β7 [F (2, 18) = 0.81, p = 0.4595] and polyubiquitinated proteins levels [F (2, 18) = 4.19, p = 0.0318]. *, p<0.05 vs. control (sham) rats. ‡, p<0.05 vs. MI-HFtr rats.

**Figure 4:**
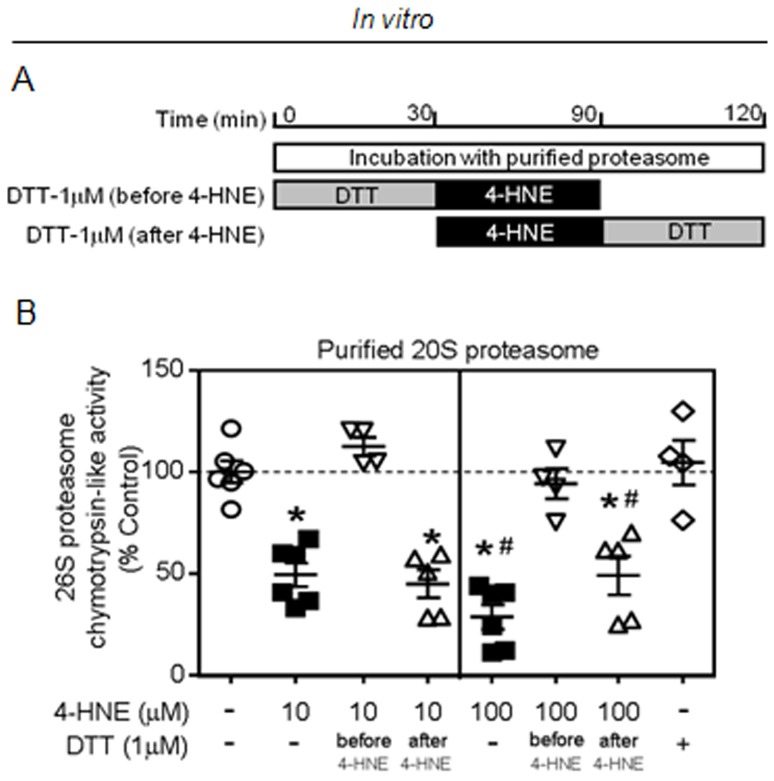
4-HNE irreversibly inactivates 20S proteasome *in vitro*. (A) Schematic panel of *in vitro* incubations. (B) Purified 20S proteasome (1 ug) was incubated for 30 min at 37°C with 4-HNE (10 or 100 µM) and proteasomal activity was measured at the end of incubation. DTT (1μ) was added to the reaction either previous or after 4-HNE incubations. Of interest, prior, but no later, incubation with DTT protected 4-hydroxi-2-nonenal inhibition of proteasomal activity. Error bars indicate SEM. Proteasomal activity [F (7, 32) = 21.37, p<0.0001]. *, p<0.05 vs. control, 4-HNE (10 µM)+DTT (before). #, p<0.05 vs. 4-HNE (10 µM).

**Figure 5:**
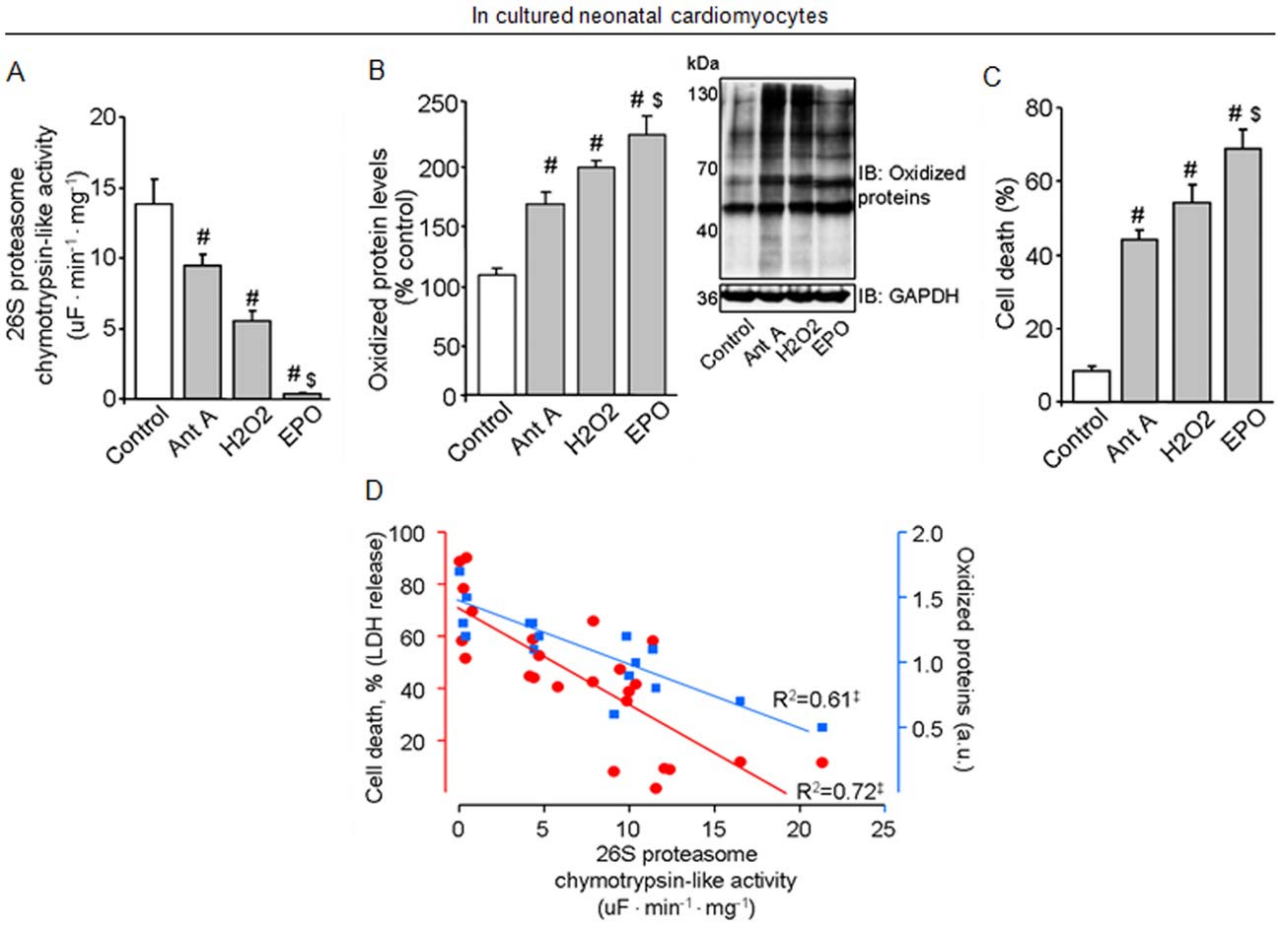
Oxidative stress contributes to proteasomal inactivation, accumulation of damaged proteins and cell death in cultured neonatal cardiomyocytes. Proteasomal activity (A), oxidized protein levels and representative blots (B) and cell death (C) in cultured neonatal cardiomyocytes. Concordance between proteasomal activity, oxidized protein levels and cell death in cultured neonatal cardiomyocytes (D). Cells were stimulated with antimycin A (100 µM, Ant A, gray bars) or H_2_O_2_ (100 µM, gray bars) or Epoxomicin (1 µM, EPO, gray bars) for 2 hours. Measurements were performed 24 hrs after treatments. Experiments were repeated at least 5 times. Protein expression was normalized by GAPDH. Error bars indicate SEM. Proteasomal activity [F (3, 20) = 30.85, p<0.0001]; oxidized protein levels [F (2, 9) = 21.84, p = 0.0003] and cell death [F (3, 14) = 27.53, p<0.0001]. #, p<0.05 vs. non-treated cells (control). $, p<0.05 vs. antimycin A- and Epoxomicin-treated cells.

**Figure 6:**
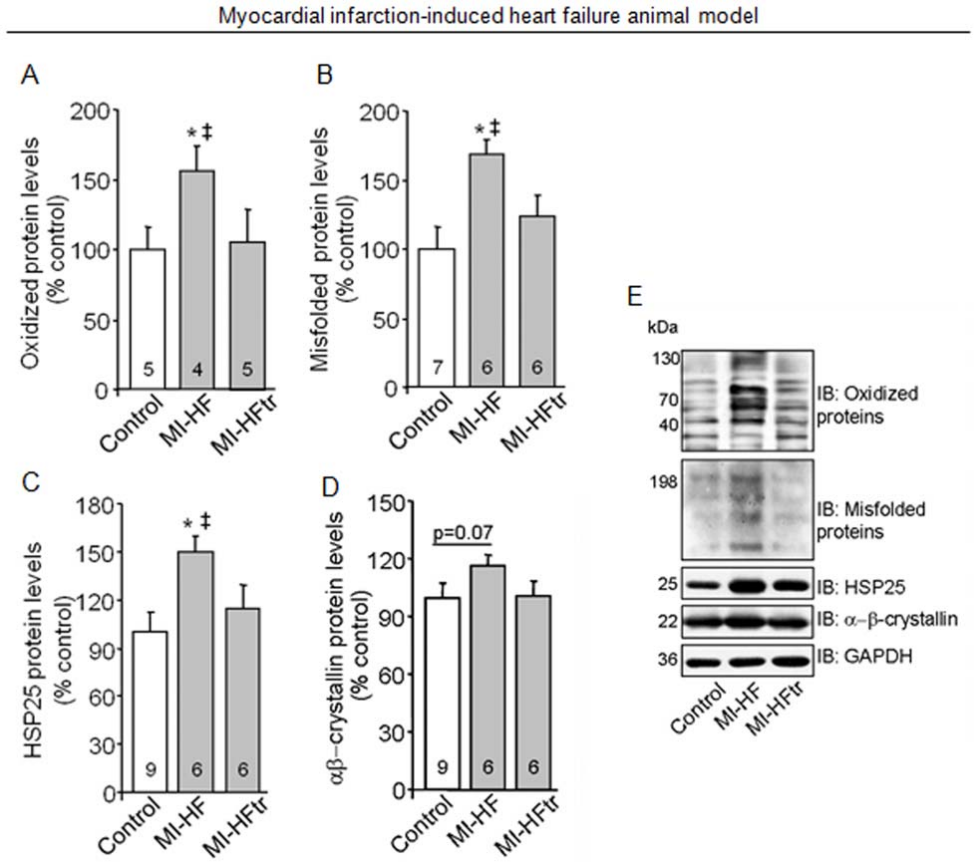
Exercise training improves protein quality control in myocardial infarction-induced heart failure. Oxidized protein levels (A), soluble oligomers accumulation (B), HSP25 (C) αβ-crystallin (D) protein levels in heart samples from 24 week-old control (sham, white bars), MI-HF (gray bars) and MI-HF exercise trained (MI-HFtr, gray bars) rats. Representative blots of oxidized protein, soluble oligomers, HSP25, αβ-crystallin and GAPDH (E). All measurements were performed in the ventricular remote area. Protein expression was normalized by GAPDH. Error bars indicate SEM. Oxidized protein levels [F (2, 19) = 5.25, p = 0.0312]; soluble oligomers accumulation [F (2, 15) = 3.97, p = 0.0412]; HSP25 [F (2, 19) = 4.21, p = 0.0306] and αβ-crystallin proteins levels [F (2, 17) = 1.49, p = 0.0252]. *, p<0.05 vs. control (sham) rats. ‡, p<0.05 vs. MI-HFtr rats.

Data are presented as means ± standard error of the mean (SEM). Data normality was assessed through Shapiro-Wilk's test and those no presenting normal distribution were log transformed (i.e. distance run and peak VO_2_ before experimental protocol, and interventricular septum in diastole before and after experimental protocol). One-way analysis of variance (ANOVA) was used to analyze data presented in Figures 2, 3, 4, 5A–C and 6. Two-way ANOVA for repeated measures was used to analyze data depicted in Figure 1 and Tables 1 and 2. Whenever significant F-values were obtained, Tukey's adjustment was used for multiple comparison purposes (p-values displayed on Tables 1 and 2 legends). Statistical significance was considered achieved when the value of P was <0.05. Linear regression was used to assess the association between variables in Figure 5D.

## Results

### Exercise training improves cardiac function and oxygen uptake in heart failure animals

At four weeks after myocardial infarction surgery (Figure 1A), heart failure rats displayed reduced cardiac function and exercise intolerance (Figures 1B–C). These changes were accompanied by a pathological cardiac remodeling, depicted by increased heart weight/body weight ratio (HW/BW), left ventricular dilation, cardiomyocyte hypertrophy and elevated cardiac fibrosis compared to control animals (Tables 1 and 2). No changes in heart rate and blood pressure were observed (Figures 1D and E).

Eight weeks of aerobic exercise training (Figure 1A) significantly increased cardiac function, depicted by elevated ventricular fractional shortening and ejection fraction in heart failure animals (Figure 1B and Table 2). These findings were accompanied by a cardiac anti-remodeling effect, characterized by reduction of both cardiac collagen deposition and left ventricular dilation (Tables 1 and 2). Exercise training had no effect on HW/BW ratio and myocardial infarction area (Table 1). Cardiomyocyte width was normalized in trained heart failure animals towards control group (Table 1). Based on the data described above, we demonstrate a switch from pathological to physiological cardiac remodeling in trained heart failure animals, since exercise training improved cardiac function along with a prominent morphological change. These findings corroborate our previous work showing that aerobic exercise training promotes a cardiac anti-remodelling effect in a sympathetic hyperactivity-induced heart failure animal model [36]. Finally, the effectiveness of exercise training was demonstrated by increased exercise capacity, peak VO_2_, citrate synthase activity and resting bradycardia in the trained heart failure animals (Figure 1C–D and Table 1).

### Failing hearts display reduced mitochondrial function and exacerbated ROS release

Impaired mitochondrial metabolism associated with increased ROS release has been shown to contribute to a number of cardiovascular diseases [7], [37]. In order to assess mitochondrial function in failing hearts, we measured oxygen consumption and maximum calcium uptake in isolated mitochondria from 24 week-old myocardial infarction-induced heart failure rats and age-matched controls (Figure 2A–D). Our results indicate that heart failure rats displayed reduced state 3 respiratory rate along with a significant decrease in the efficiency of mitochondrial oxidative phosphorylation compared to control (*sham*) animals, as measured by respiratory control ratio (State 3/State 4) (Figure 2A–C). Of interest, reduced mitochondrial efficiency was paralleled by the inability of heart failure animals to perform prolonged physical activity (Table 1). Isolated mitochondria from failing hearts also displayed impaired maximum calcium uptake (Figure 2D). Strikingly, a moderate exercise training protocol (over 8 weeks) increased both state 3 and state 4 respiratory rates as well as re-established the efficiency of mitochondrial oxidative phosphorylation (Figures 2A–C). Moreover, exercise training improved maximum calcium uptake and exercise tolerance in heart failure animals (Figures 2D and Table 1).

Considering that ROS release has been strongly associated with changes in oxygen consumption and heart failure [7], we decided to measured H_2_O_2_ release in isolated mitochondria from 24 week-old failing hearts. Our results demonstrated that H_2_O_2_ release was significantly increased in mitochondria from heart failure animals compared to age-matched control group (Figure 2E). Interestingly, exercise training decreased mitochondrial H_2_O_2_ release to control group values.

### 4-HNE modification inhibits proteasomal peptidase activity in heart failure

Elevated ROS release has been generally implicated in cellular damage during pathological processes. The introduction of carbonyl functional groups into proteins by 4-HNE, a major product of ROS-mediated lipid oxidation, has been reported to induce protein inactivation during ischemia-reperfusion injury [38]. Here, we found that non-infarcted cardiac zone from 24 week-old heart failure rats displayed exacerbated accumulation of 4-HNE-protein adducts compared to control animals (Figure 3A). Moreover, immunoprecipitation experiments showed that 4-HNE modifications of the 20S proteasome were elevated in failing hearts compared to controls (Figure 3B). Interestingly, exercise training was able to reduce the formation of adducts between 4-HNE and 20S proteasome in heart failure animals (Figure 3A–B).

Because oxidative modifications of the proteasome have been reported to reduce its proteolytic activity during ischemia-reperfusion injury [9], we decided to evaluate the efficacy of the proteasome chymotrypsin-like site (the main proteolytic site involved in peptide degradation) to cleave the artificial substrate LLVY-AMC *in vitro*. These experiments were performed in the non-infarcted cardiac zone from 24 week-old heart failure rats and their age-matched controls. As shown in Figure 3C, proteasomal activity was strikingly reduced in failing hearts compared to controls, with no changes in protein expression of 20S proteasome subunits (α5/α7, β1/β5/β7) (Figure 3D and F). Reduction of proteasomal activity was accompanied by accumulation of polyubiquitinated proteins in failing hearts (Figures 3E and F). Interestingly, exercise training re-established proteasomal activity and decreased polyubiquitinated protein levels in failing hearts. Therefore, our results suggest that exercise training prevents 20S proteasome dysfunction in heart failure, likely due to formation of 4-HNE adducts.

### In vitro 4-HNE modification inhibits proteasomal activity in an irreversible manner

To directly test the effect of 4-HNE modification on proteasome function, purified 20S proteasome was incubated with 4-HNE for 60 min at 37°C (Figure 4A). *In vitro* incubation of purified 20S proteasome with different concentrations of 4-HNE (10 and 100 µM) resulted in a significant reduction of proteasomal activity (Figure 4B). In order to test whether proteasome inhibition was mediated by oxidative modifications, purified 20S proteasome was pre-treated with DTT (1 µM). Indeed, reduction of free sulphydryl groups prior to incubation with either 10 µM or 100 µM of 4-HNE was effective to protect the proteasome against oxidative modification-mediated inhibition. Another explanation for the benefits of DTT pre-treatment might be related to a direct neutralization of 4-HNE oxidative capacity. Interestingly, DTT post-treatment of purified 20S proteasome did not rescue the proteasomal inactivation mediated by 4-HNE modifications. These results demonstrate that 4-HNE modification induces inactivation of proteolytic 20S proteasome activity *in vitro* in an irreversible manner.

### Oxidative stress contributes to proteasomal inactivation, accumulation of damaged proteins and cell death in cultured neonatal cardiomyocytes

In order to validate our *in vivo* and *in vitro* findings showing that mitochondrial dysfunction-mediated oxidative modification inactivates proteasome and results in accumulation of damaged proteins, we challenged cultured neonatal cardiomyocytes isolated from rats with either antimycin A (a mitochondrial complex III inhibitor) or H_2_O_2_. Antimycin A (100 µM) resulted in a significant reduction of *in vitro* proteolytic proteasomal activity in an ATP-independent manner, since ATP was added during *in vitro* measurements (Figure 5A). Antimycin A-mediated proteasomal inhibition was accompanied by accumulation of oxidized proteins and elevated cardiomyocytes death (Figures 5B–C). These findings demonstrate that mitochondrial dysfunction-mediated ROS release inactivates proteasome and impairs removal of damaged proteins. Indeed, treatment of cardiomyocytes with either H_2_O_2_ (100 µM) or Epoxomicin (1 µM, a selective proteasome inhibitor) drastically reduced proteasomal activity along with accumulation of oxidized proteins and cell death (Figures 5A–C). Finally, we found a tight correlation between proteasomal inactivation, accumulation of oxidized proteins and cell death in cultured neonatal cardiomyocytes treated with antimycin A, H_2_O_2_ or Epoxomicin (Figure 5D).

### Proteasomal inhibition disrupts cardiac protein quality control in heart failure rats

The ubiquitin proteasome system is the primary effector of the protein quality control process in cardiomyocytes [39]. Considering our *in vivo* and cell cultured findings demonstrating that mitochondrial dysfunction mediates inactivation of proteasome and accumulation of damaged proteins, we evaluated the cardiac protein quality control profile in 24 week-old myocardial infarction-induced heart failure rats.

Heart failure animals displayed significant accumulation of oxidized proteins compared to age-matched controls (Figure 6A and E). In order to test whether decreased proteasomal activity would contribute to misfolded proteins accumulation, we evaluated cardiac soluble pre-amyloid oligomers levels in heart failure rats. In fact, myocardial infarction-induced heart failure rats presented a significant increase of misfolded proteins levels compared with control hearts (Figure 6B and E). Moreover, heart failure rats presented a significant increase in HSP25 protein levels compared to controls (Figure 6C and E). Our findings showing inhibition of the proteasome along with accumulation of damaged proteins and increased expression of small chaperones demonstrate a clear disruption of protein quality control in failing hearts. Interestingly, exercise training reduced cardiac oxidized and misfolded proteins levels in heart failure rats compared with age-matched non-trained heart failure animals (Figure 6A–B and E). This profile was accompanied by a reduction of small chaperone levels in trained animals (Figure 6C and E), suggesting that improved proteasomal activity mediated by aerobic exercise training contributes, at least in part, to better cardiac protein quality control in heart failure.

## Discussion

Over the past decades, significant progression has been made in understanding the cellular processes involved in heart failure, which has positively contributed to drug development in this field. However, in spite of new therapies able to improve patient's quality of life and survival [1], heart failure remains the main cause of death worldwide. Thus, there is a compelling need for new pharmacological and non-pharmacological therapies that could improve clinical outcomes. To fulfil this issue, a number of studies have focused on identifying intracellular distal strategic nodes where signals converge and/or serve as multi-effector brakes to suppress or reverse heart failure, which would become attractive targets for heart failure therapy.

Due to its pivotal role in bioenergetics, calcium homeostasis, redox regulation and cell death, mitochondria have been considered an intracellular organelle capable of orchestrating biochemical processes across the cell [40]. Indeed, much of the current research focuses on understanding the crosstalk between mitochondrial and the rest of the cell. In the present study, we found that mitochondrial dysfunction-associated 4-HNE accumulation, a highly reactive α,β-unsaturated aldehyde and a major secondary product of lipid peroxidation, contributes to protein quality control disruption by directly targeting the proteasome in failing hearts. Moreover, we demonstrated that *in vitro* 4-HNE-modification/inactivation of proteasome occurs in an irreversible manner, while reduction of free sulphydryl groups prior to 4-HNE incubation abolished the inhibition of proteasomal chymotrypsin-like activity. Our study has shown for the first time that modification/inactivation of the cardiac proteasome by the lipid peroxidation product 4-HNE occurs in heart failure. Others have observed the same phenomenon during acute coronary occlusion/reperfusion, cerebral ischemia and aging [9], [18].

The proteasome has been implicated in the removal of polyubiquitinated and oxidatively modified proteins [41], [42], [43], [44]. Therefore, impairment of proteasomal proteolytic activity by 4-HNE may negatively affect cellular protein quality control and further contributes to cell death. In agreement with these findings, we observed a striking inactivation of proteasomal chymotrypsin-like activity paralleled by accumulation of oxidatively modified, misfolded and polyubiquitinated proteins in failing hearts. These responses were accompanied by increased expression of small chaperones. Over-expression of small chaperones such as HSP25 and αβ crystallin is related to cellular protection against misfolded protein accumulation [45] and cell death [46] under acute insults (i.e. acute cardiac ischemia-reperfusion injury). However, during chronic degenerative diseases such as heart failure, increased chaperones expression does not overcome the deleterious effects generated by accumulation of misfolded proteins.

Considering that maintenance of protein quality control is crucial to protect long-lived cells, such as cardiomyocytes and neurons, we evaluated whether mitochondrial dysfunction-mediated oxidative stress could affect proteasomal activity and overall protein quality control in cultured isolated cardiomyocytes. Either Antimycin A or H_2_O_2_ resulted in proteasomal inactivation, accumulation of oxidatively modified proteins and cell death in cultured cardiomyocytes. These findings demonstrate that oxidative stress-mediated chymotrypsin-like proteasomal inhibition (the main proteasomal proteolytic site involved in protein degradation and the most sensitive to 4-HNE modification) [10] decreases cardiomyocyte viability and contributes, at least in part, to the disruption of cardiac protein quality control in cardiomyocytes. We have previously shown that improvement of proteasomal activity using pharmacological tools protects neonatal cardiomyocytes against H_2_O_2_-induced cell death [42].

Emerging studies have revealed that disruption of cardiac mitochondrial metabolism and/or proteasomal insufficiency are not only implicated but also play an important role in cardiac pathogenesis. In fact, selective pharmacological and genetic therapies capable to rescue either mitochondrial metabolism or proteasomal activity improve cardiac function in different heart disease animal models [39], [42], [47], [48]. However, considering that both mitochondrial metabolism and proteasomal function are highly regulated by different cellular processes, we cannot exclude the possibility that these changes are secondary to the compromised cardiac function. Therefore, further studies investigating both direct and indirect proteasomal regulation by oxidative stress during heart failure progression are required. Also, the contribution of oxidative stress to other proteolytic systems such as autophagic/lysosomal pathways, and its effect on cardiac protein quality control in HF should be considered.

The possible mechanisms involved in 4-HNE-mediated proteasomal inhibition have been previously described in different cell lines and systems, including the heart. Analysis of two-dimension gel electrophoresis from purified proteasomes followed by mass spectrometry demonstrated that 4-HNE modification is not a random process and that specific proteasomal subunits, mainly α-subunits, are targeted by 4-HNE in the heart [9], [10], [49]. However, since the proteasomal catalytic sites are located in the β subunits (β1, β2 and β5), it has been suggested that an indirect mechanism, probably mediated by protein-protein interactions between regulatory and catalytic subunits, drives the inhibition of proteasomal peptidase activity mediated by 4-HNE. Indeed, further studies are required to better clarify this issue.

Another important finding of this study is the efficacy of exercise training in restoring cardiac mitochondrial function, proteasomal activity and protein quality control in heart failure animals. Exercise capacity has been widely recognized an independent predictor of mortality in patients with cardiovascular diseases [24]. Moreover, exercise training is considered an important adjuvant in the treatment of heart failure since it increases both peak VO_2_ and exercise tolerance, resulting in improved patient outcome and quality of life [4], [5], [6]. However, the mechanisms underlying exercise-induced beneficial effect on heart failure are not completely understood.

Over the last decades, several studies have demonstrated the contribution of exercise training to improve expression of mitochondrial markers of biogenesis and metabolism in heart failure. However, the contribution of exercise training to mitochondrial physiology and its extension to cytosolic systems related to cell survival during heart failure remains unclear. A recent study has demonstrated that low-intensity interval exercise training decreases calcium-induced mitochondrial permeability transition in aortic-banded miniature swine [2], which may positively affect cytosolic systems. In addition, exercise training has been shown to improve cardiac redox balance in young and old healthy animals [23], [50]. We extended these findings by showing that 8 weeks of aerobic exercise training restored oxidative phosphorylation efficiency along with a reduction in H_2_O_2_ release and increased maximum calcium uptake in isolated mitochondria from myocardial infarction-induced heart failure rats. Interestingly, exercise training had a positive impact on cytosolic protein quality control machinery by re-establishing the proteasomal activity in failing hearts. These findings suggest that reduced cardiac oxidative stress along with better protein quality control are associated with the benefits promoted by exercise training in heart failure rats. However, considering the complexity of mitochondrial metabolism and protein quality control machinery, further investigations need to be conducted in order to establish a cause-and-effect relationship as well as clarify other possible regulatory mechanisms regulated by exercise training in heart failure.

In summary, we provide evidence that myocardial-infarction induced heart failure rats display a prominent cardiac mitochondrial dysfunction, 4-HNE accumulation and cytosolic protein quality control disruption. In addition, the ability of exercise training to rescue mitochondrial function, decrease 4-HNE accumulation and improve cardiac protein quality control in heart failure highlights an important molecular mechanism underlying the benefits of exercise training in failing hearts.
